# Supplementary material for: Exploring Black Soybean Extract Cream for Inflammatory Dermatitis—Toward Radiation Dermatitis Relief
Source: Int J Mol Sci. 2024 Oct 29;25(21):11598. doi: 10.3390/ijms252111598 (PMC11546988; doi:10.3390/ijms252111598)
Supplement: Supplementary file 1 [file ijms-25-11598-s001.zip › ijms-3287014-supplementary.pdf]

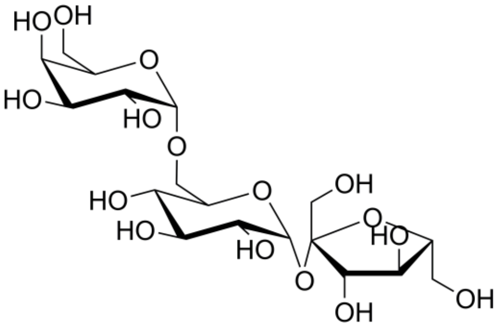

Raffinose

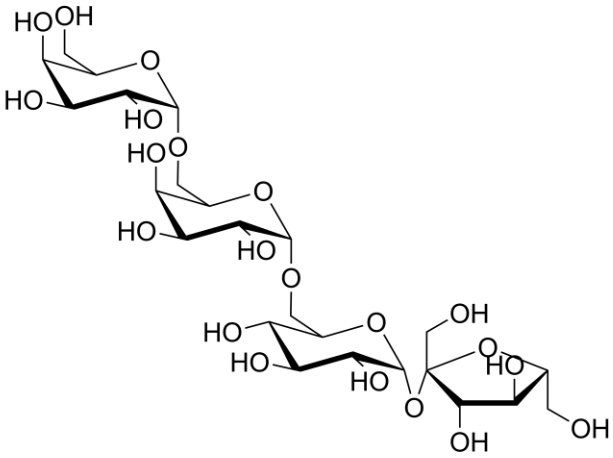

Stachyose

Supplemental Figure S1: Structures of Raffinose and Stachyose.

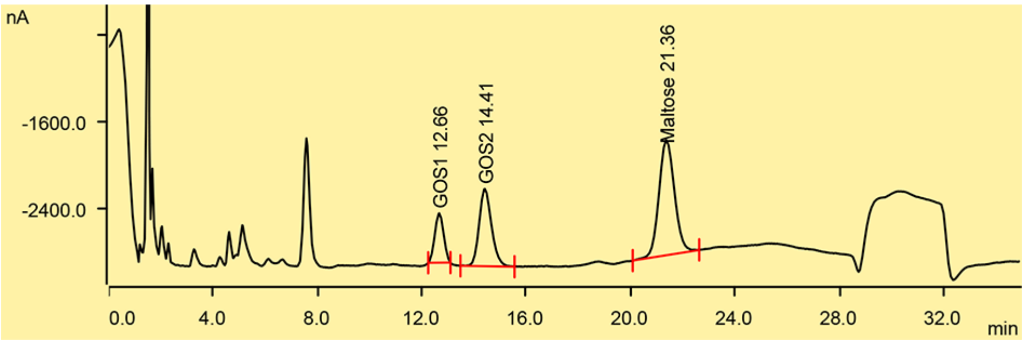

Supplemental Figure S2: The ion chromatogram of a sample. GOS1 denotes Raffinose and GOS2 denotes Stachyose. Please note: Maltose was used as an internal standard for the analysis of Raffinose and Stachyose and was not an active ingredient in the cream.

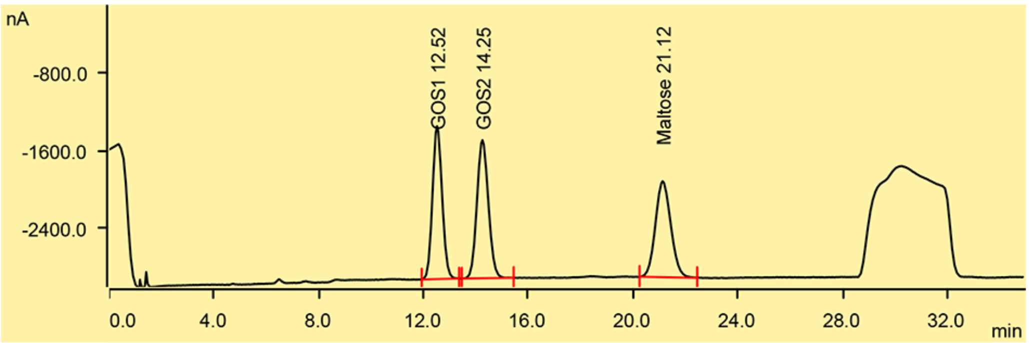

Supplemental Figure S3: The ion chromatogram of GOS1 and GOS2 standard. GOS1 denotes Raffinose and GOS2 denotes Stachyose. Please note: Maltose was used as an internal standard for the analysis of Raffinose and Stachyose and was not an active ingredient in the cream.
